# An Estimate of the Numbers and Density of Low-Energy Structures (or Decoys) in the Conformational Landscape of Proteins

**DOI:** 10.1371/journal.pone.0005148

**Published:** 2009-04-09

**Authors:** Kanagasabai Vadivel, Gautham Namasivayam

**Affiliations:** Centre of Advanced Study in Crystallography & Biophysics, University of Madras, Tamilnadu, India; Dalhousie University, Canada

## Abstract

**Background:**

The conformational energy landscape of a protein, as calculated by known potential energy functions, has several minima, and one of these corresponds to its native structure. It is however difficult to comprehensively estimate the actual numbers of low energy structures (or decoys), the relationships between them, and how the numbers scale with the size of the protein.

**Methodology:**

We have developed an algorithm to rapidly and efficiently identify the low energy conformers of oligo peptides by using mutually orthogonal Latin squares to sample the potential energy hyper surface. Using this algorithm, and the ECEPP/3 potential function, we have made an exhaustive enumeration of the low-energy structures of peptides of different lengths, and have extrapolated these results to larger polypeptides.

**Conclusions and Significance:**

We show that the number of native-like structures for a polypeptide is, in general, an exponential function of its sequence length. The density of these structures in conformational space remains more or less constant and all the increase appears to come from an expansion in the volume of the space. These results are consistent with earlier reports that were based on other models and techniques.

## Introduction

Current theories of protein folding postulate an energy landscape for the polypeptide chain that always includes a ‘folding funnel’, with the native conformation at its bottom [1], [2]. The chain has an initial random (self-avoiding) conformation, and then, during the folding process, follows multiple paths down the funnel to attain the final folded conformation [3], [4]. It is also postulated that the sequence of the polypeptide has evolved such that the folding pathways offer minimal frustration [5], [6]. Thus, not only is the final conformation at the bottom of the funnel distinctly the preferred equilibrium state, but it is also the one most easily attained from any random starting conformation [7]. These design principles thus allow only a small portion of the unimaginably vast sequence space to be actually possible in biological systems [2], [7].

Despite these limitations on the sequences, for each one of them a large number of low-energy structures can be computed using all known models of the interactions that drive the folding process [8]–[11]. These structures are often referred to as decoys, and have potential energy values comparable to, or even more favourable than the native, experimentally determined structure [12], [13]. The decoy structures mimic many of the other characteristics of true protein structures, such as the secondary structure content, the numbers of native contacts, and the possession of a hydrophobic core. However they are not biologically active conformations. The general inability to unambiguously and consistently distinguish between the decoys and the native structure is one of the weaknesses of current theories and methods in protein folding, as well as in *ab initio* protein structure prediction [14].

Decoy structures are not merely an expression of the errors in the model force fields. They could be kinetic traps in the folding pathway that, under appropriate – perhaps pathological – conditions, lead to misfolded structures [3]. They could be intermediate states in the folding pathway, between the molten globule state and the native state, which are populated transiently, slowing down the folding process, eventually to converge on the native structure [15], [16].

Several workers have generated libraries of decoy structures that serve primarily to test and refine theories and methods of protein structure prediction [8], [9]. These include discrete state models [12] as well as off-lattice models [17]. The methods used to generate the decoy sets include Monte Carlo optimisation with simulated annealing [18], random search of conformational space with subsequent local minimization [10], molecular dynamic trajectories [19]–[21], the graph theoretic algorithm [22] and a fragment insertion method using Bayesian scoring functions [23]. Park and Levitt [12] used a highly simplified model to generate decoy sets of 35,000 to 200,000 decoy structures each for eight proteins. Using a novel relaxation method to further refine this set, they ended up with a library of decoys consisting of 650 structures on an average for each protein. Keasar and Levitt [10] generated decoy set for 14 small proteins with different folds using random sampling of conformational space with subsequent local energy minimization. They started with 100,000 structures for each protein. After refinement the final data set consists of about 450 decoys for each protein. Using an all-atom model [9], Tsai and his group generated decoys for 78 diverse proteins with different topologies. The final library contains about 1400 decoys for each protein. Apart from the above decoy sets, such sets have also been constructed for loop structures in proteins. The one constructed by Samudrala and Moult [24], consists of 400 decoys per loop.

In all methods mentioned here, the final number of decoys has been selected from about 100,000 starting structures. The exact number of initial structures generated for each protein depended on the computational costs involved, and ranged from as low as 26 [18] to as high as 14,000 [21]. It is not clear from the literature if these numbers were based on estimates of the numbers of decoy structures that possibly exist in the conformational space of a protein.

Based on a random energy model, Bryngelson and Wolynes [25] have earlier made an estimate of the possible numbers of metastable structures, of which the ones with the deeper minima would correspond to folding intermediates. They found that the numbers of such structures increase as [exp(αn)]/n, where α is of the order of 1 and n is the number amino acid residues. A more recent estimate, which was based on computations of all-against-all gapless threading amongst a database of 1,011 non-homologous proteins, with an optimised potential of interactions [26], showed that for current models and force fields, proteins of length 150 to 250 residues could have about 10^12^ decoys distinct from the native structure, distributed uniformly over the conformational space.

Here, we report our estimates of the exhaustive numbers of possible decoys (low-energy structures) that exist for a given sequence. These estimates are made using the MOLS algorithm and the ECEPP/3 force field [27]. We have shown that this algorithm, which was developed in our laboratory [28], has the ability to identify all the low energy conformers of a given peptide sequence. We apply this method to exhaustively identify the numbers and densities of low-energy structures in the conformational space of several peptides, ranging in length from 5 to 10 residues. We then extrapolate the results to estimate these numbers for protein sequences of any length.

## Methods

### The use of mutually orthogonal Latin squares (MOLS) in exploring conformational space

As detailed elsewhere [28] the technique uses MOLS to perform an unbiased and exhaustive conformational search to locate minimal energy conformations of a peptide. In the design of agricultural or clinical experiments [29] MOLS sampling is used to reduce the size of the experimental space. If m is the number of variable parameters in the experiment, or in other words, the number of dimensions in the experimental space, and n is the number of points along each dimension, the size of the experimental space is n^m^ points. MOLS are used to identify a sample of size m^2^ points of these n^m^ points, without serious loss of information. Thus, to identify the optimal point in the space, instead of performing n^m^ experiments, only m^2^ experiments are performed. This sample is then statistically analyzed to obtain optimal point. In our application of this technique, we cast the problem of conformational search to identify optimal (low energy) conformations as one of experimental design, and use MOLS to identify a small sample as representative of the vast conformational space. For reasons made clear below, however, we cannot use same techniques of analyses of variance to analyse this sample. Instead we use a variant of the mean field technique to analyse the sample of conformational space selected by MOLS and use that to identify the optimal conformation. Thus it would be appropriate to explain the calculations from the viewpoint of the mean field technique (MFT).

MFT has been previously used to address conformational search problems [30], [31], for example to arrive at the optimal side-chain configurations of a protein, given a specific back-bone fold. Here we use this application as an example to elucidate the technique. If Φ is the search space (for example, all possible side chain conformations), this is divided into a number of subspaces ϕ_i_ (for example, individual side chains). Each such subspace has a number of states ϕ_ij_ (for example, the side chain rotamers), each with a probability of occurrence ρ_ij_. The effective potential due to a state ϕ_rs_ of a subspace ϕ_r_ is given as
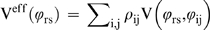
(1)where the summation is over all subspaces i ≠ r, and all the states of these subspaces. V(ϕ_rs_, ϕ_ij_) is the interaction potential between ϕ_r_ and ϕ_i_, calculated with ϕ_r_ set to ϕ_rs_, and ϕ_i_ set to ϕ_ij_. The procedure starts by assigning ρ_ij_ = 1/m_i_ (m_i_ is the number of states of the subspace ϕ_i_, all equally probable), and equation (1) is used to evaluate the effective potential for all states of all subspaces. The probabilities ρ are re-evaluated as
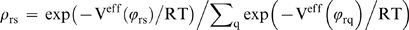
(2)where the summation is over all the states of the subspace r. R is the gas constant and T is the temperature. The newly determined probabilities are then used to re-determine the effective potentials. This is iterated until the probabilities converge to a set of self-consistent values. The set of most probable states of the subspaces defines the most probable state of the system (e.g. the most probable set of side-chain conformations).

In applying this technique to the conformation of peptides, (and not just the side chains) we define the subspaces as the torsion angles (including the backbone torsion angles), and the states as the values that these angles can assume. Once again, initially all values are equally probable, and the effective potential due to setting the torsion angle ϕ_r_ to the value ϕ_rs_ is calculated using equation (1). However, this extension to torsion angle space is not straightforward, since the potential V in the summation cannot be calculated by simply considering the two torsion angles ϕ_r_ and ϕ_i_ alone – we need to set all other torsions also to specific values. In other words, the interaction between a pair of subspaces (when the subspaces are the torsion angles) does not depend only on their respective states, but depends also on the states of all other subspaces. The expression V(ϕ_rs_, ϕ_ij_) is thus not sensible in this context, and the summation in equation (1) has to be performed over all possible combinations of the states of all the subspaces except ϕ_r_. This will clearly lead to combinatorial explosion since the number of such combinations is an exponential function of the number of subspaces.

To overcome this problem, we use a small sample of the possible combinations, in other words, a small sample of the conformational space, to calculate the effective potential. We use mutually orthogonal Latin squares (MOLS) to perform this sampling. The procedure to construct a set of MOLS is given in the supplementary material (Text S1) along with a schematic diagram (Figure S1 and Figure S2). If there are n such torsion angles, each with m possible values, then ϕ_rs_, (r = 1, n; s = 1, m) defines the search space in which the sampling is to be carried out. To calculate the effective potential due to setting ϕ_r_ to ϕ_rs_ we now use the following equations.
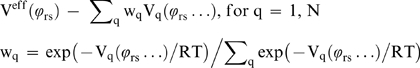
(3)


The summation is over all the N points in the MOLS grid at which ϕ_r_ is set to ϕ_rs_. V_q_ is the potential function. The ellipsis in the expression for the potential indicates the setting of all the other torsion angles (except ϕ_r_), determined using the MOLS algorithm. V^eff^(ϕ_rs_) is used to evaluate the probability of the value ϕ_rs_ for ϕ_r_ – the value with the lowest effective potential being the most probable. The set of most probable values for the angles defines the low energy conformation of the peptide. It may be noted that in this formulation the procedure is no longer an iterative one. The weights w_q_ in equation (3) are not the same as the probabilities ρ in equation (2). Thus one cycle of MOLS calculations leads to one low energy conformation. To locate another low energy structure, we perform another cycle of calculations, again selecting m^2^ points in the conformational space using a different set of MOLS. For n subspaces with m states each, there are (m!)^n^ different ways of choosing a set of MOLS [32]. Using any one of them as the basis for one cycle of calculations would lead to a low energy structure. The procedure may be repeated several times, with different sets of MOLS, to eventually identify all the low energy conformations. We have earlier [33] demonstrated that the procedure is exhaustive. Since this is an important point in the present discussions, we shall do so again here, in the ‘Results’ section. A stepwise presentation of the algorithm is given in supplementary figure S3.

### Clustering

To ensure an exhaustive sample, we used the MOLS procedure to generate 10000 structures for each selected peptide sequence. However every cycle of the procedure does not lead to a new structure, and very often the structure obtained at the end of one run is the same as, or similar to, another generated by a different run. Thus, the next step in the procedure is to weed out the similar structures in the sample, and restrict the library to only the unique ones. This was accomplished by use of the following clustering procedure. The first structure generated was placed in the first bin. The second generated structure was compared to the first by least squares superposition and calculation of the rmsd in atomic coordinates. If the rmsd was less than a specific value, the second structure was considered the same as the first one, and placed in the same bin. If not it was placed in a new bin. If there were more than one structure in a bin, subsequent comparisons were made between all the structures in that bin and newly generated structure. If the new structure had an rmsd of less than a specified cut off value with any one of the members in a bin, it was placed in that bin. The new structure was then compared with the structures in all remaining bins, placed in every bin in which it found a match. The procedure was repeated for all 10,000 structures generated for each sequence.

Many structures had rmsd less than the cut-off value with many other structures and thus appeared in multiple clusters. The procedure therefore incorporated a second pass to ensure that each structure appeared in only one cluster. This was accomplished as follows. Assume, for example, that structure number one appeared in many bins. In each such bin, this structure was compared to every other structure in that bin, and an average rmsd calculated. The structure was then assigned to the bin in which it had the lowest average rmsd, and deleted from all the other bins. This procedure was repeated for all structures in multiple bins, until finally all the 10,000 generated structures were sorted into a smaller number of clusters, and each structure appeared in only one cluster. The centroid of each cluster was then recalculated, and used to represent that cluster. The above procedure is similar to the one adopted by Betancourt and Skolnick [34].

The number of clusters, or equivalently, the number of unique structures, and the number of structures in each cluster varied according to the length of the sequence as well as the choice of rmsd cut-off. This is discussed in greater detail in the ‘Results’ section.

### Potential energy landscape of the decoys

We used principal coordinate analysis [35] to visualize the potential energy landscape of the decoy structures of the sequences. We achieved this by projecting the full multi-dimensional space on an appropriate low-dimensional sub-space, where the variance of projection is maximized along orthogonal directions. The procedure operates on the n×n distance matrix, which is based on a similarity measure between any two conformations. As in the clustering, the coordinate based rmsd is used as a similarity measure. This distance matrix is transformed into a centred matrix, which is then diagonalized. The resulting eigenvalues are normalized to give the percentage of the projection of the original distribution on the new set of axes. The eigenvectors, scaled by their corresponding eigenvalues, give the coordinates of the original data points in the new axes. The best two principal coordinates, i.e., the ones that explain the largest portions of the total variance in the data set, are used to view the energy landscape.

### Sequence selection

We selected six sequences with lengths 5, 6, 7, 8, 9 and 10 residues, respectively, for the calculations. The sequences were all chosen from the PDB [36] so that the experimental structure could provide a point of reference for the calculations. Of the six, three are free-standing peptides. Since there were no such sequences in the PDB of lengths 6, 7 and 9, peptides of these three lengths were chosen from loop sequences in larger proteins. The selected loop sequences are found more than once in the PDB, and they have the same structure in all the occurrences – the root mean square distance between the backbone atoms when they appear in different proteins is <1 Å in all three cases. We therefore consider that these loop sequences have an independent structure, irrespective of the rest of the protein. There were other sequences that also had these properties, but these three are the best in this category, and have better resolution, R-factors and average temperature factors than the others.

### Choice of potential energy function

Several potential energy functions have been used in modelling protein structures [27], [37], [38]. Some of these are statistical functions, constructed by mining the structure databases [23], [39]–[41]. In the present calculations, since we are mapping the energy landscapes of peptides, we used the ECEPP/3 [27] potential energy function. We did not use any explicit solvent terms. As reported earlier [42], and as may be seen from the results, the function performs well enough to identify low energy structures very similar to the experimental ones, indicating its suitability. In order to ensure that the results were not only a consequence of the choice of the potential function, we repeated the calculations with the AMBER [38] force field as well. It may be noted that discussions of protein folding pathways usually refer to the free energy [43], [44]. However, it is common to calculate potential energy maps and use these to discuss the energy landscapes [33], [35].

## Results

The results are presented in four parts. In the first part we establish that the MOLS algorithm carries out an exhaustive search of conformational space. In the second one we count the number of decoy structures for sequences of various lengths, and extrapolate from these results to longer sequences. In the third we calculate the densities of the decoys in conformational space. In the final section we sketch out the six energy landscapes considered and describe their features.

### The search is exhaustive

**Figure 1:**
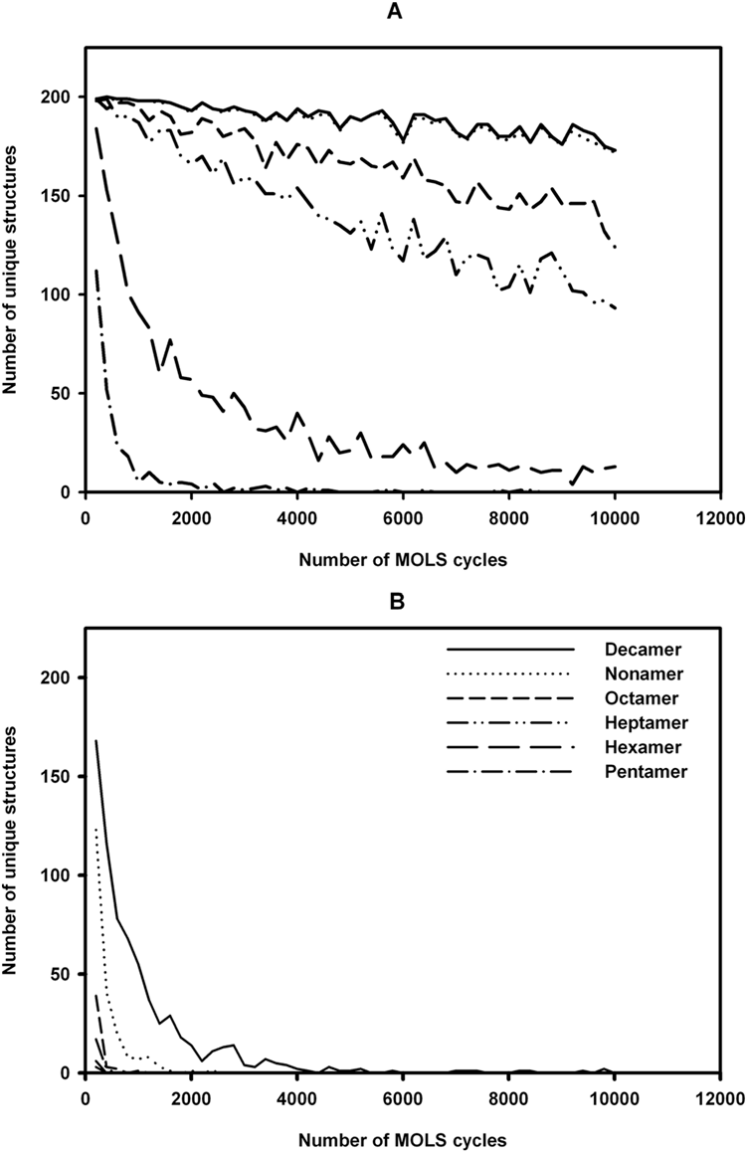
The MOLS sampling is exhaustive. The number of unique structures identified at different rmsd cutoff values in 10,000 MOLS cycles with the ECEPP/3 potential for each peptide. A : At 1.0 Å rmsd cutoff. B: At 2.0 Å rmsd cutoff.

We establish this in three different ways. Firstly, Figure 1A shows that as the number of structures generated increases, the number of new (or unique) structures falls off sharply. This is especially clearly seen in the case of the smaller peptides, though the trend is clear even for the larger ones. Understandably, the number of unique structures depends on the rmsd cut-off used in the clustering process. At a cut-off value of 1.0 Å, for the pentapeptide, all the unique structures have been identified by the 8303 structure generation cycle, and no new ones are identified after this. For the decapeptide, at the same cut-off, new structures are discovered even after 10,000 structures have been generated, though the number of such structures being discovered begins to drop. If the cut-off is increased to 2.0 Å, for the pentapeptide, almost all the 10,000 structures generated are the same; for the decapeptide, no new structures appear after generation number 9788 (Figure 1B). Thus, it is clear that the algorithm exhaustively searches the conformational space, given a sufficient number of iterations. Though the number of iterations required rapidly increases with the size of the peptide, this reflects the number of such structures actually present in the conformational landscape, and is not a limitation of the algorithm.

**Figure 2:**
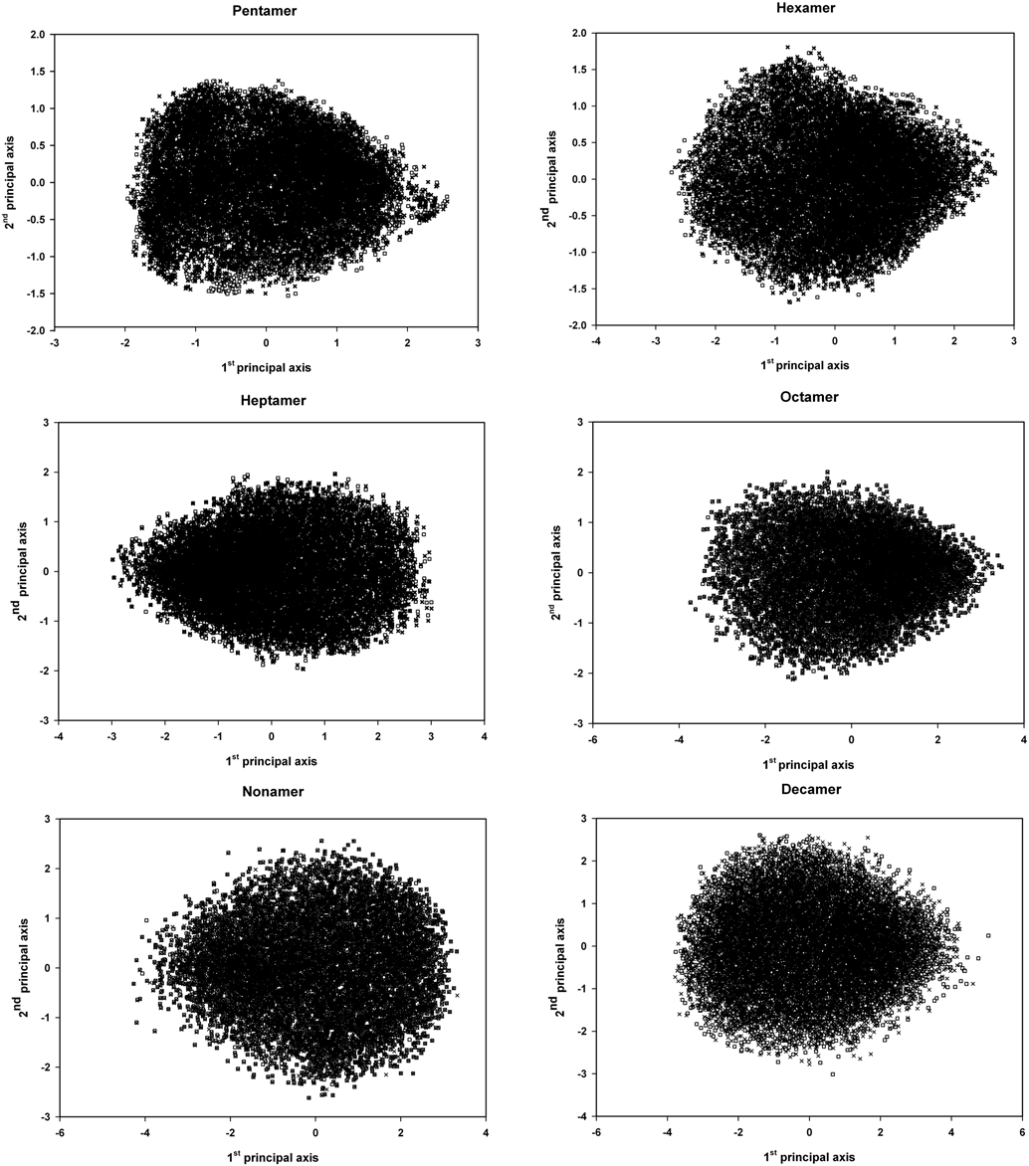
Joint projection of the conformational samples. The joint projection of two different conformational samples on the first two principal coordinates for each peptide. Crosses represent conformations from the first sample; squares represent conformations from the second. The two samples cover exactly the same area in the low-dimensional principal sub-space, indicating that each conformation sample covers the entire available conformation space.

The second corroboration for the exhaustive nature of the search comes from an application of the sample overlap procedure [35] to the structures generated. According to this procedure, two independent conformational samples of the same system are generated using different protocols (e.g. different initial conditions or different initial random number seeds). If the two samples overlap and occupy the same area in conformational space, the sampling is exhaustive. We have generated two samples of 10,000 structures each for the pentapeptide and the decapeptide. Figure 2 shows the joint projections of the two samples on the first two principal coordinates for each peptide, calculated as described in the methods section. Clearly, in both cases, the two samples cover exactly the same area in the low-dimensional principal sub-space, thus establishing that MOLS sampling exhaustively covers the conformational space of the molecules.

**Figure 3:**
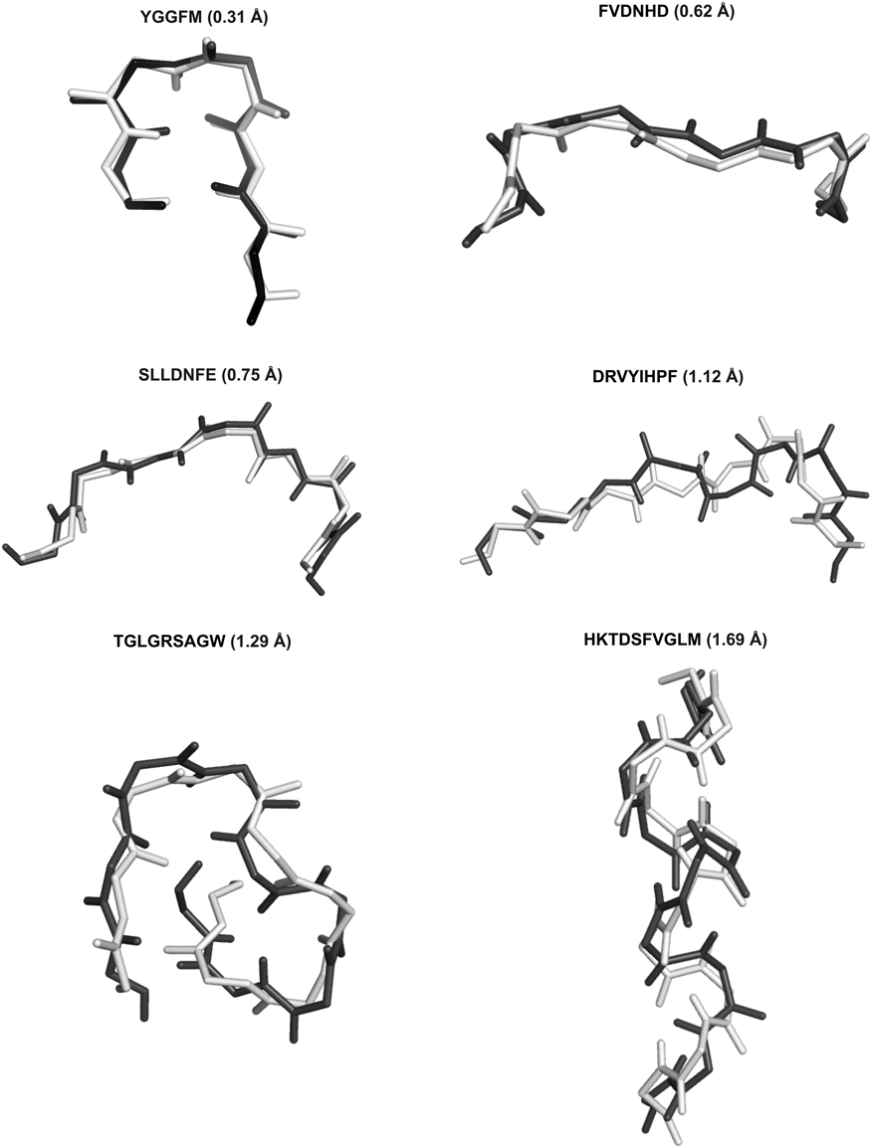
Comparison with experimental structures. The best matched, i.e. lowest rmsd structure (black) superposed on the respective crystal structure (white) for each peptide. Only the backbone atoms are shown.

Finally, Table 1 (and Figure 3) gives a comparison of the generated structures with the respective experimental structure. For each peptide, some of the former are accurate replicas of the latter, and have very low values of the rmsd in atomic positions on superposition. This is true for the loop sequences as well, though the force field did not use any information about the flanking sequences, or about the interactions the residues in the loop make with the rest of the protein or with the atoms of the solvent. In addition, as we have discussed elsewhere [42], the MOLS search also identifies other low energy-structures observed by other techniques, both experimental and theoretical. For example, in the case of the neuropeptide Met-enkephalin the sample of 1500 structures contained the global energy minimum as revealed by other calculations [45]–[47], besides the structures seen in experiments [48]. Again these facts support our contention that, despite the relatively small number of structures generated, MOLS sampling covers conformational space thoroughly. In general, the energy values of the structures closest to the experimental results are not the lowest of all the generated structures. However they are within about 25 kcal/mol of the latter, with no short contacts or other unphysical interactions.

**Table 1:** Quality of the MOLS sampling.

Sequence Length	Sequence	(A)	(B)	Best Predicted structure		Lowest Energy structure	
				rmsd (Å)	Energy (kcal/mol)	rmsd(Å)	Energy (kcal/mol)
5	YGGFM	2.04	−9.61	0.30	0.04	3.73	−15.30
6	FVDNHD	2.68	−45.63	0.62	−37.57	3.00	−56.89
7	SLLDNFE	2.74	−47.50	0.75	−41.88	3.45	−60.45
8	DRVYIHPF	3.1	−53.32	1.12	−44.93	4.19	−70.58
9	TGLGRSAGW	3.45	−39.23	1.29	−38.05	2.96	−55.07
10	HKTDSFVGLM	4.01	−22.11	1.69	−29.96	4.25	−38.3

Comparison between the structures generated using MOLS and the respective experimental structures. When the structures were binned according to their energy values, the ones in the lowest 10% bin were compared with the experimental structures. Column (A) gives the average rmsd of this set, and column (B) gives the average energy of the structures in this set. The other columns compared the single best predicted structure and the one with the lowest energy with the respective experimental structures.

### How many low-energy structures (decoys) are there in conformational space?

**Figure 4:**
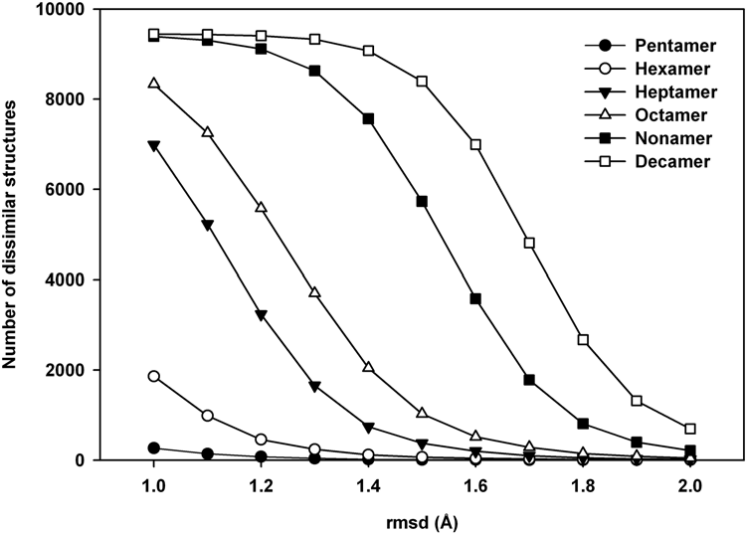
Unique structures identified with ECEPP/3 force field. The number of mutually dissimilar structures found at different rmsd cutoffs for each peptide in 10,000 MOLS structures using the ECEPP/3 potential.

Figure 4 gives the total number of mutually dissimilar, or ‘unique’, structures that remain after clustering the 10,000 generated structures at different cut-off values. As explained in the ‘Methods’ section, a high value for the rmsd cut-off would result in a lower number of unique structures after clustering, and vice versa. The unique structures give an indication of the number of low-energy structures, or decoys, that are present in the conformational space of the peptides. Thus, if the clustering is carried out at a cut-off of 1 Å, the pentapeptide has 265 low energy structures, while the decapeptide has more than 9000 such conformations. At 2.0 Å cut-off, these numbers reduce to just 3 for the pentamer, and to less than 700 for the decamer. Thus, at all the rmsd cut-off values applied, every sequence considered has a limited number of low-energy structures.

**Figure 5:**
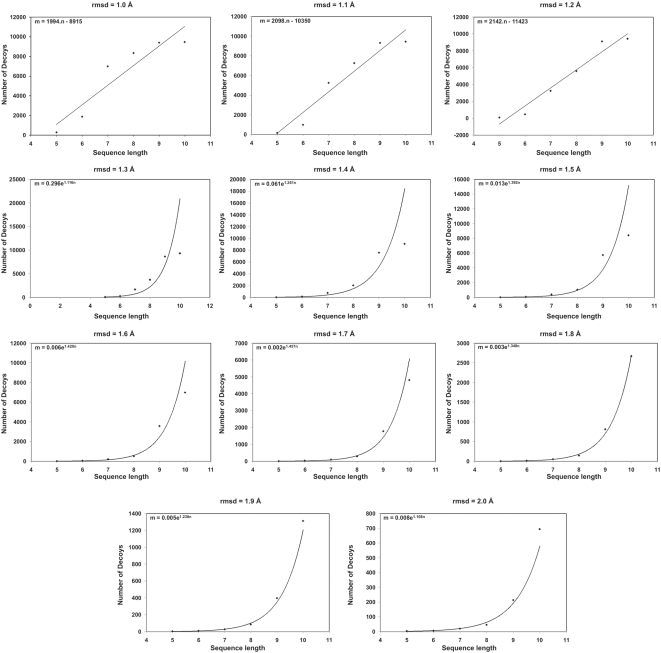
Density of decoy structures. The number of decoys at various rmsd cut-off values as a function of sequence length.

This number increases with the length of the sequence. Figure 5 is plot of the number of decoys at various cut-off values as a function of sequence length. At low cut-off values, (less than 1.3 Å), the increase is linear. If the trend at 1.0 Å is extrapolated, one may expect 30965 decoys to populate the conformational space of a 20 mer, and 40935 decoys that of a 25 mer.

At high cut-off values (greater than 1.3 Å), the increase is exponential. If the trend at 2.0 Å is extrapolated, a 20-mer would have 3.36×10^7^ decoys in its conformational space and a 25-mer would have 8.57×10^9^ decoys. The general expression in this case to calculate the number of decoys m as a function of the sequence length n, is m = a×exp(bn), where a is of the order of 1/n and b is of the order of 1.0. These results tally remarkably well with those of Bryngelson and Wolynes [25]. An attempt was made to calculate the number of decoys for a 20 mer as well as for a 25 mer, to verify that the number fit these results. The sequences chosen were the loop sequences AGNSGYSQGTIGYPGALPNA from the structure of the protein Sphericase [49] (PDBID 1EA7_A167-186) and AGKSSDSKGIDLTNVTLPDTPTYSK, from the structure of inorganic pyrophosphatase [50] (PDBID 1E9G_A231-255). Even at rmsd cut-off values as high as 2.6 Å, all 10,000 structures generated for each sequence were mutually dissimilar, and clustering did not reduce the total number of low energy structures. Strictly speaking, this result is compatible with both a linear increase, as well as with an exponential increase. However, it is probable that at least some of the 10,000 structures generated would be similar to each other, if the totals were to be about 31,000 and 41,000 structures for the 20-mer and 25-mer respectively as predicted by the linear model. Since this is not the case in the present calculations, these results further indicate that the increase is exponential.

**Figure 6:**
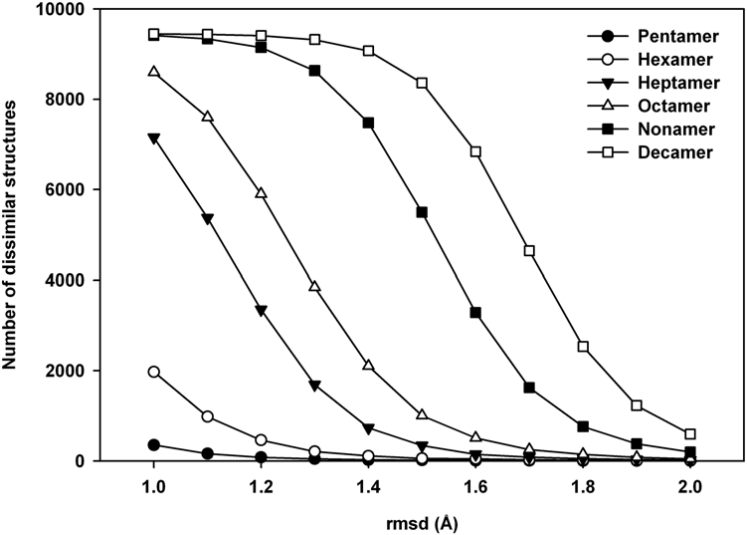
Unique structures identified with AMBER force field. The number of mutually dissimilar structures found at different rmsd cutoffs for each peptide in 10,000 MOLS structures using the AMBER force field.

To evaluate the effect of the force field, all the calculations were repeated with the AMBER potential function [38]. The results were remarkably similar to those obtained above with ECEPP/3. The numbers of unique structures identified for each sequence length with AMBER parameters at different rmsd cut-off values are shown in Figure 6. The plot is a replica of Figure 4. Once again, the increase in the number of low energy structures at lower rmsd cutoff (<1.3 Å) is linear, while at larger cutoff (>1.3 Å) the number increases exponentially with sequence length. We thus conclude that the conformational landscape of a protein consists of approximately exp(n) low energy structures, or decoys, where n is the sequence length.

### Density of the decoys

In calculating the numbers of decoy structures above, we have made the implicit assumption that these structures are uniformly distributed in conformational space, and that their density, i.e. the number of unique structures per unit conformational volume, is approximately independent of the sequence or sequence length. This assumption may also be stated as follows. The increase in the number of unique structures with sequence length is due to the increase in the total conformational volume of the larger molecule, and not due to any increase in the number of unique structures per unit conformational volume. We attempted to test this assumption by calculating the density as the ratio of the number of structures to the conformational volume. Of the two sets of values required here, the former has been estimated above. However the latter, i.e. the volume, is not simply a function – an exponential function – of the number of the degrees of freedom of the molecule. This is apparent when we consider that two structures may be separated quite substantially in a conformational space parameterized in terms of the torsion angles, but may yet have an rmsd in their atomic positions less than the cut-off used to estimate the number of decoys. We have therefore estimated the volume of the conformational space available to each molecule in terms of the area projected by the entire set of decoys of each molecule on the respective first two principal coordinate axes. Principal coordinate analyses transforms a complex multidimensional space to another orthogonal set of axes, such that the first principal coordinate reflects the largest portion of the structural variance in the data set, the second one the next largest, and so on. In the present case, the transformation yielded sets of principal axes, the first two of which accounted for more than 25.5% of the variance in each of the six data sets considered. Figure 2 indicates the nature of these projections. We used the areas of these projections as representative of the volumes of the respective conformational spaces, and have calculated the densities on this basis.

Table 2 gives the projected area, the number of structures in this area and the calculated density for each of the six sequences. As expected, the projected area increases with sequence length, reflecting the behavior of the total conformational volume. If we use the 10,000 structures generated for each sequence to calculate the densities, the pentamer has the highest density, while the decamer has the least density. This is obviously due to the increase in the projected area, while maintaining the number of structures constant. However, if we use only the unique structures (at cut-off 1.0 Å), the density does not show any pattern related to sequence length, but is approximately the same for all the molecules, about 1000 structures per Å^2^ in rmsd. In other words, for any given low energy structure, there are about 1000 other low energy structures within rmsd of 1 Å. At larger rmsd cut-off (2.0 Å) the smaller sequences have very few structures, and it is difficult to estimate the density from our data.

**Table 2:** Density of the decoys.

Sequence Length	Projected Area (Å^2^)	Density (All structures)	At 1.0 Å rmsd cutoff	
			Number of structures used	Density
5	10.31	969.93	265	25.70
6	14.65	682.59	1860	126.96
7	17.84	560.54	6988	391.70
8	23.37	427.90	8332	356.53
9	32.69	305.90	9390	287.24
10	38.87	257.27	9441	242.89

The densities (in units of structures/Å^2^) of decoy structures in the conformational landscapes at different sequence lengths as calculated from the PCoorA analysis. The projection area is calculated using the formula π*(a/2)*(b/2), where ‘a’ and ‘b’ are the largest distances between the points along the first and second principal axes, respectively.

### Energy landscapes of the decoys

**Figure 7:**
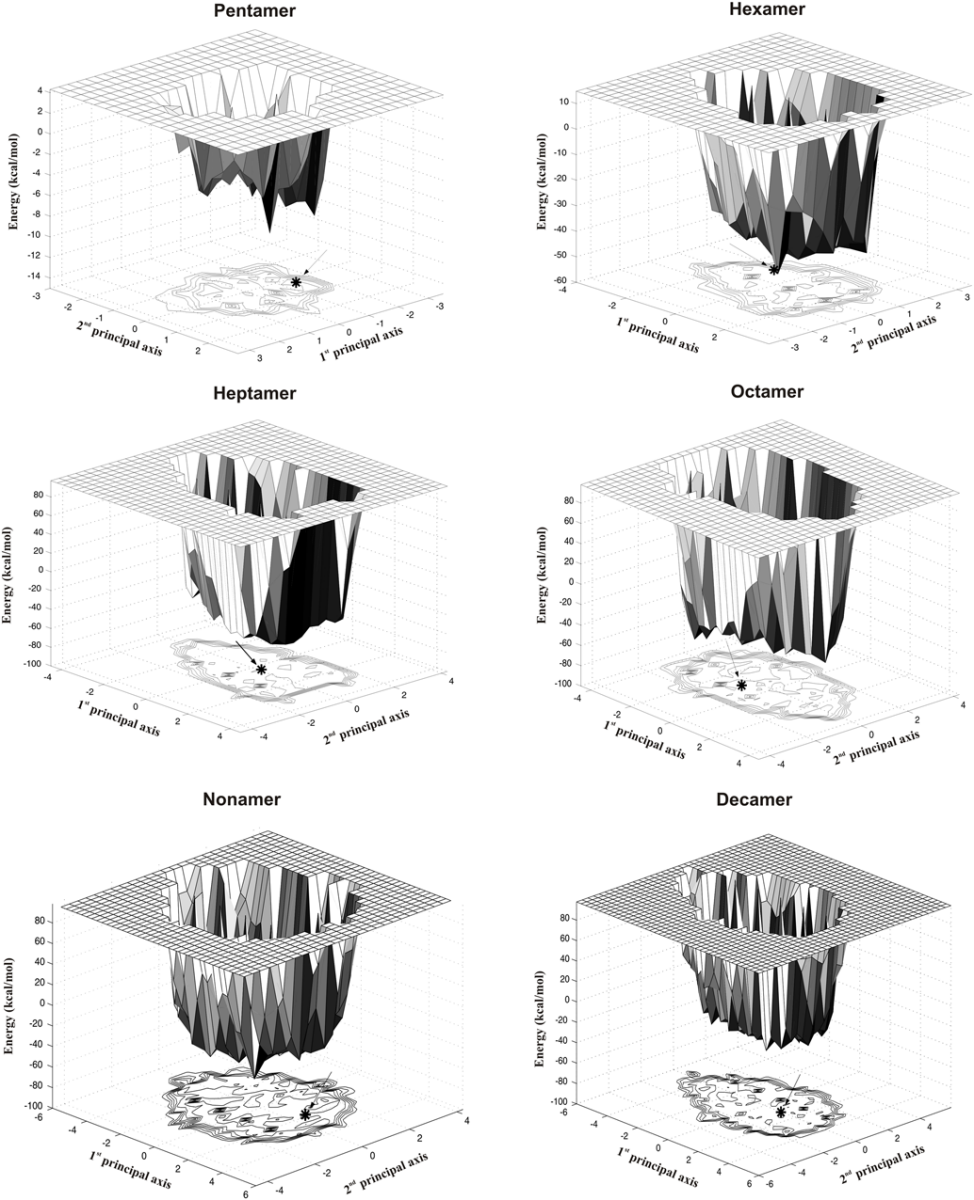
Energy landscapes of the peptides. Energy landscaps drawn at 1.0 Å rmsd cut-off. Arrow points to the position of the experimental structure.

**Figure 8:**
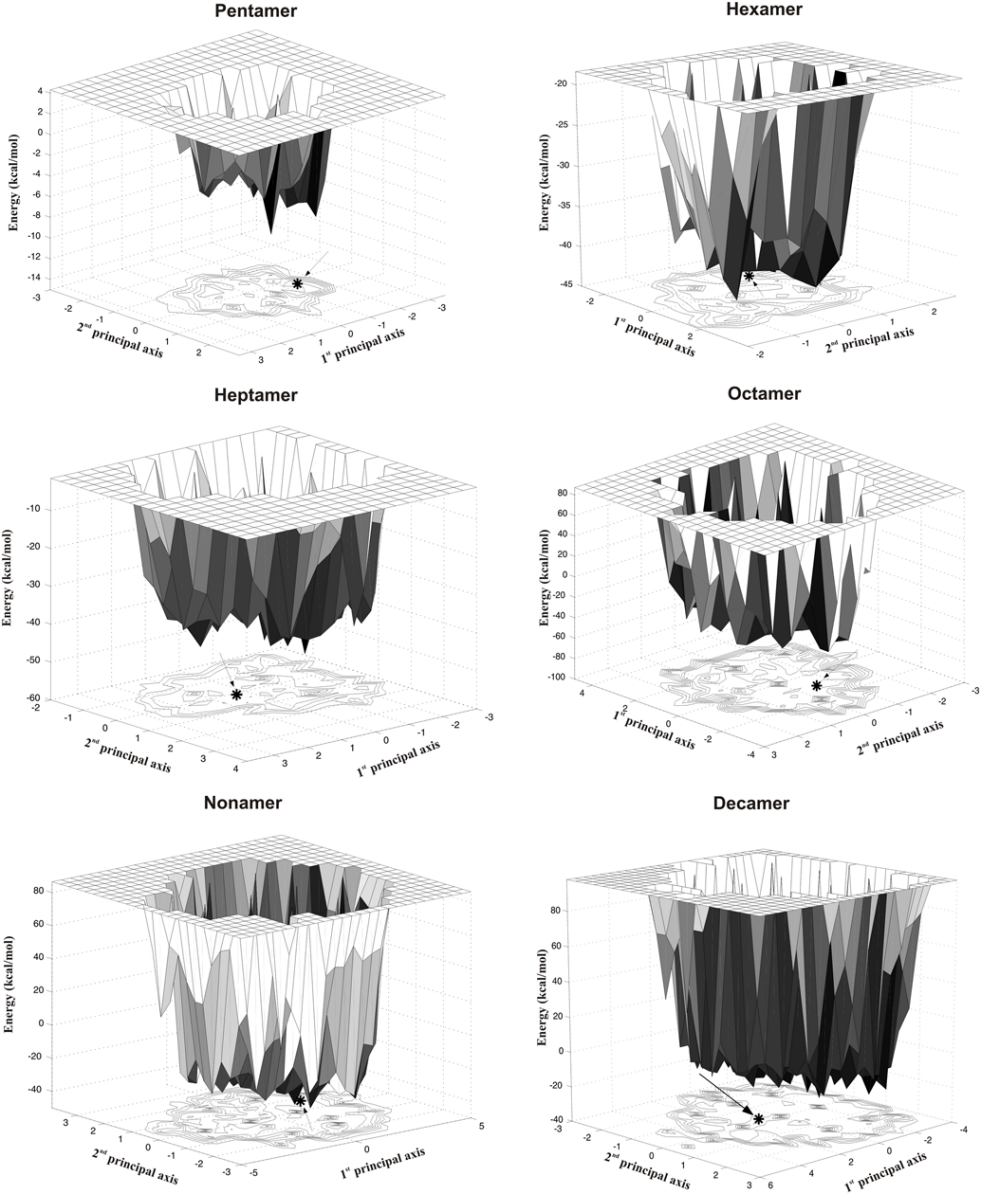
Energy landscapes of the peptides. Energy landscaps drawn at different rmsd cutoffs (see text for details). Arrow points to the position of the experimental structure.

As mentioned earlier in the paper, energy landscape theory postulates the existence of a deep, rather narrow, native well in the energy landscape of a protein. The native or near-native structures at the bottom of the well have energies significantly lower than those in the vicinity. Following the work of Levy and Becker [35], we have used the MOLS-generated structures to visualize the conformational energy landscape of the molecules by projection on the space of the first two principal coordinates. Figure 7 shows these landscapes, drawn using the unique structures obtained by clustering at 1.0 Å cut-off. Pictured at low resolution, the landscapes are more or less featureless, and the experimental structure (marked by an asterisk on the projection plane) is not clearly distinguishable from the rest of the structures. This is especially true for the larger sequences. Thus structures far apart in conformational space have the same energy. The landscapes may also be viewed at a higher resolution. For this view we used a more stringent cut-off, based on the overall structural similarities of the 10,000 structures generated, and calculated separately for each sequence as follows. The structures are clustered at 1.0 Å cut-off. For each cluster an average rmsd is calculated by comparing all the structures within the cluster with each other. The average values are then again averaged over all the clusters, and half this value is taken as a suitable cut-off. All 10,000 structures are then clustered using this cut-off, and the set of unique structures so obtained were used to draw the landscapes (Figure 8). This procedure avoids the use of arbitrary cut-off values, and allows the comparison of the landscapes of molecules of different sizes on an approximately equal footing. Figure 8 shows the portion of the landscape immediately surrounding the experimental structure for each sequence. It is clear that the ruggedness increases with increasing sequence length. Though the overall topologies of the landscapes are similar, for each sequence there are clearly a few structures that have distinctly lower energy than all the others, and one of these lower energy structures is in most cases the native experimental structure. However, in general, more than fifty percent of the structures generated are within 5 kcal/mol of the experimental ones in energy. Note that though the structures are close in energy, they are quite different in conformation. When the structures were clustered into different energy bins, those in the bin containing the lowest energy structures had rmsd between 2 Å and 5 Å as compared to the other members of the bin.

## Discussion

**Figure 9:**
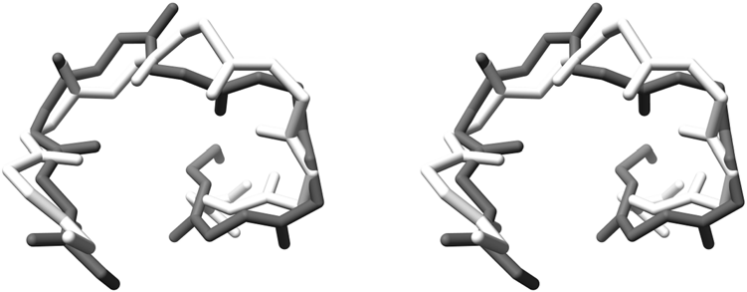
Performance of MOLS with explicit water. Stereo diagram of the best identified model (black) with the explicit water molecules for the nonameric sequence TGLGRSAGW, superimposed with its respective crystal structure (white).

**Figure 10:**
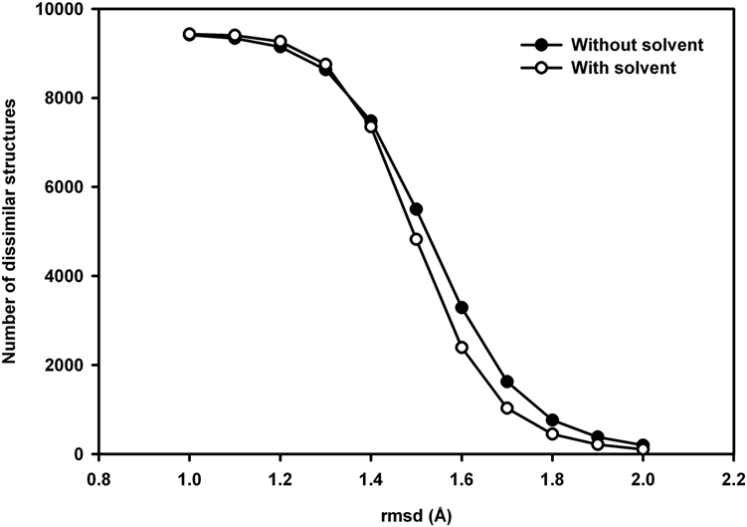
Unique structures identified with and without hydration. Comparison of the number of dissimilar structures found at different rmsd cut-off for a nonameric sequence by performing the sampling with and without explicit water molecules.

The results indicate that the energy function that we have used does not distinguish easily between the native structure and the decoys. Previous reports [12], [17], [51], [52], including some of our own [42], [53], have indicated that this is true of almost all known potential functions, though functions specifically designed to model a particular class of proteins (or peptides) [27], or functions based on known protein structures [39] tend to perform better than general, physics-based functions such as the one we have used. The inclusion of solvent effects has been reported to improve the identification of the native structure. Most potentials include such effects implicitly, for example in selecting the parameters defining the semi-emipirical force fields. In order to evaluate if the inclusion of explicit solvent molecules in calculating the structures would make a difference, we carried out the calculations with explicit water molecules, using the AMBER force field, for the nonameric sequence TGLGRSAGW. Besides the peptide intramolecular non-bonded terms, the force field also include the interactions between water and the peptide. Of the 10000 structures generated by the MOLS technique, the one shown in Figure 9 had the lowest rmsd of 1.82 Å with the respective native structure. The lowest energy structure showed a large deviation from the native structure (4.61 Å). The number of unique structures identified after inclusion of explicit solvent shows a pattern similar to that of in-vacuum simulations (Figure 10). This suggests that the results reported above for the vacuum simulations do not change on inclusion of explicit solvent.

In summary, an exhaustive search of the conformational landscape of peptides ranging in size from 5 to 10 residues using the MOLS sampling technique has resulted in the identification of all the low energy or decoy structures for each. The number of such structures increases exponentially with the sequence length, in consonance with previous results. The density of structures in the conformational space remains about the same irrespective of sequence length, with all the increase in the number coming from an increase in the volume of the conformational space. The energy landscapes of the peptides indicate that the native, experimental structure is not easily identifiable as the minimum energy structure in the space. Decoy structures far removed in conformation from the native structure possess comparable energy values. These results have the following implications for *ab initio* protein structure prediction. Firstly, available *ab initio* energy functions are probably not accurate enough to identify the native structure from the population of decoys. Though in the current report we have demonstrated this only for the ECEPP/3 and the AMBER force fields, other reports [12], [17], [51], [52] including some from our laboratory [42], [53], indicate similar results for other force fields and scoring functions. Secondly, the view of the energy landscape as consisting of a ‘folding funnel’ with the native structure at the bottom model has been formulated in the free-energy framework [1], [2], in which temperature is an important determination of the shape of the energy landscape of a protein. The MOLS technique, on the other hand, maps the potential energy landscape and shows that numerous low energy ‘valleys’, that could act as kinetic traps at specific temperatures, exist throughout conformational space. Thus any linear search algorithm for the native structure is unlikely to be successful, even granting a reliable method of recognizing the optimum. Thirdly, owing the presence of an extremely rugged fine structure at the bottom of the landscape, high resolution prediction of protein structure is likely to be more complex by many orders of magnitude than prediction of the overall fold.

Finally, to revisit a statement made in one of our first papers describing this method [28], one of the obstacles to applying the MOLS technique to *ab initio* protein structure prediction was the non-availability of an appropriate potential function with a deep and fairly wide minimum in conformational space corresponding to the native structure. While this remains a problem, our current results indicate that using MOLS to perform exhaustive sampling of the conformational space to pick up all possible native-like structures, (in order to identify the native structure from them) is computationally too expensive for all but the smallest of proteins.

## Supporting Information

Text S1 The procedure to construct a set of MOLS.
(0.03 MB DOC)

Figure S1 A Latin square of order 3. (a) The Latin alphabets a, b, and c and (b) the Greek alphabets α, β and γ are used as symbols for the construction of the Latin squares.
(0.15 MB DOC)

Figure S2 Two mutually orthogonal Latin squares (MOLS) of order 3. This is obtained by the super position of the two Latin squares given in Figures ‘S1a’ and ‘S1b’. Note that every symbol of the first square occurs once, and exactly once, with every symbol of the second square.
(0.08 MB TIF)

Figure S3 Flowchart of the MOLS algorithm.
(0.95 MB TIF)

## References

[1] Bryngelson JD, Onuchic JN, Socci ND, Wolynes PG (1995). Funnels, pathways, and the energy landscape of protein folding: A synthesis.. Proteins: Struct Funct Genet.

[2] Dill KA, Chan HS (1997). From Levinthal to pathways to funnels.. Nat Struct Biol.

[3] Wolynes PG, Onuchic JN, Thirumalai D (1995). Navigating the folding routes.. Science.

[4] Dinner AR, Karplus M (1999). The thermodynamics and kinetics of protein folding: A lattice model analysis of multiple pathways with intermediates.. J Phy Chem B.

[5] Weissman JS (1995). All roads lead to Rome? The multiple pathways of protein folding.. Chem Biol.

[6] Goldbeck RA, Thomas YG, Chen E, Esquerra RM, Kliger DS (1999). Multiple pathways on a protein-folding energy landscape: Kinetic evidence.. Proc Natl Acad Sci U S A.

[7] Onuchic JN, Luthey-Schulten Z, Wolynes PG (1997). Theory of Protein Folding: The Energy Landscape Perspective.. Annu Rev Phy Chem.

[8] Samudrala R, Levitt M (2000). Decoys ‘R’ Us: a database of incorrect conformations to improve protein structure prediction.. Protein Sci.

[9] Tsai J, Bonneau R, Morozov AV, Kuhlman B, Rohl CA (2003). An improved protein decoy set for testing energy functions for protein structure prediction.. Proteins: Struct Funct Genet.

[10] Keasar C, Levitt M (2003). A novel approach to decoy set generation: designing a physical energy function having local minima with native structure characteristics.. J Mol Biol.

[11] Gilis D (2004). Protein decoy sets for evaluating energy functions.. J Biomol Struct Dyn.

[12] Park B, Levitt M (1996). Energy functions that discriminate X-ray and near native folds from well-constructed decoys.. J Mol Biol.

[13] Petrey D, Honig B (2000). Free energy determinants of tertiary structure and the evaluation of protein models.. Protein Sci.

[14] Bonneau R, Baker D (2001). Ab initio protein structure prediction: Progress and prospects.. Annu Rev Biophy Biomol Struct.

[15] Sosnick TR, Mayne L, Hiller R, Englander SW (1994). The barriers in protein folding.. Nature Struct Biol.

[16] Plotkin SS, Onuchic JN (2002). Understanding protein folding with energy landscape theory. Part I: Basic concepts.. Quart Rev Biophy.

[17] Samudrala R, Xia Y, Levitt M, Huang ES (1999). A combined approach for ab initio construction of low resolution protein tertiary structures from sequence.. Pac Symp Biocomput.

[18] Holm L, Sander C (1992). Evaluation of protein models by atomic solvation preference.. J Mol Biol.

[19] Wang Y, Zhang H, Li W, Scott RA (1995). Discriminating Compact Nonnative Structures from the Native Structure of Globular Proteins.. Proc Natl Acad Sci U S A.

[20] Huang ES, Subbiah S, Tsai J, Levitt M (1996). Using a hydrophobic contact potential to evaluate native and near-native folds generated by molecular dynamics simulations.. J Mol Biol.

[21] Herges T, Wenzel W (2005). Free-energy landscape of the villin headpiece in an all-atom force field.. Structure.

[22] Samudrala R, Moult J (1998). A graph-theoretic algorithm for comparative modeling of protein structure.. J Mol Biol.

[23] Simons KT, Kooperberg C, Huang E, Baker D (1997). Assembly of protein tertiary structures from fragments with similar local sequences using simulated annealing and Bayesian scoring functions.. J Mol Biol.

[24] Samudrala R, Moult J (1998). Determinants of side chain conformational preferences in protein structures.. Protein Eng.

[25] Bryngelson JD, Wolynes PG (1987). Spin Glasses and the Statistical Mechanics of Protein Folding.. Proc Natl Acad Sci U S A.

[26] Mirny LA, Finkelstein AV, Shakhnovich EI (2000). Statistical significance of protein structure prediction by threading.. Proc Natl Acad Sci U S A.

[27] Nemethy G, Gibson KD, Palmer KA, Yoon CN, Paterlini G (1992). Energy parameters in polypeptides. 10. Improved geometrical parameters and nonbonded interactions for use in the ECEPP/3 algorithm, with application to proline-containing peptides.. J Phy Chem.

[28] Vengadesan K, Gautham N (2003). Enhanced sampling of the molecular potential energy surface using mutually orthogonal Latin squares: Application to peptide structures.. Biophy J.

[29] Finney DJ (1955). Experimental design and its statistical basis..

[30] Olszewski KA, Piela L, Scheraga HA (1992). Mean field theory as a tool for intramolecular conformational optimization. 1. Tests on terminally-blocked alanine and met-enkephalin.. J Phy Chem.

[31] Koehl P, Delarue MA (1995). Self consistent mean field approach to simultaneous gap closure and side-chain positioning in homology modeling.. Nature Struct Biol.

[32] Liu CL (1968). Introduction to Combinatorial Mathematics..

[33] Vengadesan K, Gautham N (2004). Energy landscape of Met-enkephalin and Leu-enkephalin drawn using mutually orthogonal Latin squares sampling.. J Phy Chem B.

[34] Betancourt MR, Skolnick J (2001). Finding the needle in a haystack: educing native folds from ambiguous ab initio protein structure predictions.. J Comput Chem.

[35] Levy Y, Backer OM (2001). Energy landscapes of conformationally constrained peptides.. J Chem Phy.

[36] Berman HM, Westbrook J, Feng Z, Gilliland G, Bhat TN (2000). The Protein Data Bank.. Nucl Acid Res.

[37] Brooks BR, Bruccoleri RE, Olafson BD, States DJ, Swaminathan S (1983). CHARMM: A program for macromolecular energy, minimization, and dynamics calculations.. J Comput Chem.

[38] Weiner SJ, Kollman PA, Nguyen DT, Case DA (1986). An all atom force field for simulations of proteins and nucleic acids.. J Comput Chem.

[39] Sippl MJ (1995). Knowledge-based potentials for proteins.. Curr Opin Struct Biol.

[40] Samudrala R, Moult J (1998). An all-atom distance-dependent conditional probability discriminatory function for protein structure prediction.. J Mol Biol.

[41] Melo F, Sanchez R, Sali A (2002). Statistical potentials for fold assessment.. Protein Sci.

[42] Vengadesan K, Gautham N (2004). MOLS - A program to explore the potential energy surface of a peptide and locate its low energy conformations.. Biopoly.

[43] Plotkin SS, Onuchic JN (2000). Investigation of routes and funnels in protein folding by free energy functional methods.. Proc Natl Acad Sci U S A.

[44] Hardin C, Eastwood MP, Prentiss M, Luthey-Schulten Z, Wolynes PG (2002). Folding funnels: The key to robust protein structure prediction.. J Comput Chem.

[45] Li Z, Scheraga HA (1987). Monte Carlo-Minimization Approach to the Multiple-Minima Problem in Protein Folding.. Proc Natl Acad Sci U S A.

[46] Scheraga HA, Lee J, Pillardy J, Ye YJ, Liwo A (1999). Surmounting the Multiple-Minima Problem in Protein Folding.. J Glob Optimiz.

[47] Floudas CA, Klepeis JL, Pardalos PM, Farach-Colton M, Roberts FS, Vingron M, Waterman M (1999). In DIMACS series in discrete mathematics and theoretical computer science..

[48] Griffin JF, Langs DA, Smith GD, Blundell TL, Tickle IJ (1986). The Crystal Structures of [Met5]enkephalin and a Third Form of [Leu5]enkephalin: Observations of a Novel Pleated β Sheet.. Proc Natl Acad Sci U S A.

[49] Almog O, Gonzalez A, Klein D, Greenblatt HM, Braun S (2003). The 0.93Å crystal structure of sphericase: a calcium-loaded serine protease from Bacillus sphaericus.. J Mol Biol.

[50] Heikinheimo P, Tuominen V, Ahonen AK, Teplyakov A, Cooperman S (2001). Toward a quantum-mechanical description of metal-assisted phosphoryl transfer in pyrophosphatase.. Proc Natl Acad Sci U S A.

[51] Felts AK, Gallicchio E, Wallqvist A, Levy RM (2002). Distinguishing native conformations of proteins from decoys with an effective free energy estimator based on the OPLS all-atom force field and the Surface Generalized Born solvent model.. Proteins: Struct Funct Genet.

[52] Lazaridis T, Karplus M (1999). Discrimination of the native from misfolded protein models with an energy function including implicit solvation.. J Mol Biol.

[53] Kanagasabai V, Arunachalam J, Prasad PA, Gautham N (2007). Exploring the conformational space of protein loops using a mean field technique with MOLS sampling.. Proteins: Struct Funct Bioinf.

