# Supplementary material for: An Estimate of the Numbers and Density of Low-Energy Structures (or Decoys) in the Conformational Landscape of Proteins
Source: PLoS One. 2009 Apr 9;4(4):e5148. doi: 10.1371/journal.pone.0005148 (PMC2663821; doi:10.1371/journal.pone.0005148)
Supplement: Text S1 — The procedure to construct a set of MOLS. (0.03 MB DOC) [file pone.0005148.s001.doc]

In this supplemental material, we give the pseudo code used for the construction of MOLS. As mentioned in the methods section, we use mutually orthogonal Latin squares (MOLS) to perform this sampling. This is an adaptation of a technique employed in experimental design [1] that uses MOLS to reduce the sampled search space to m^2^ points (or n^2^ points, if n is greater than m, where n is the number of subspaces, and m is the number of states for each).

A Latin square (LS) of order N, is an arrangement of N symbols in a square of side N, such that each symbol occurs exactly once in each row and once in each column. Two LS are orthogonal, if, on superposition, every symbol of the first square occurs once, and exactly once, with every symbol of the second square. Figures ‘S1a’ and ‘S1b’ show two LS of order 3. Figure S2 shows the resultant pair of orthogonal Latin squares, when they are superposed. A set of n mutually orthogonal Latin squares (MOLS) is formed when we have n Latin squares, every pair of which is orthogonal. To use MOLS to systematically sample the conformational space of peptides, we map the symbols to the values of the variable torsion angles in the molecule. Thus, with reference to the example in Figure S2, x, y and z would represent possible values for one torsion angle in a ‘molecule’ that has only two variable torsion angles, and *α*, *β* and *γ* would represent the possible values of the other. We will label the sub squares by the indices u = 1, N and t = 1, N and use the symbol φ_r,u,t_ to specify the value of the r^th^ torsion angle as found in the sub square given by the index pair (u, t). To each φ_r,u,t_ we assign a value chosen from the set θ_r,s_  by putting

φ_r,u,t_ = θ_r,s_

for all values of r = 1, n; s = 1, N and t = 1, N. The index u is calculated for each set of values of r, s and t by

u = [(t-1)(r) + (s-1)]modulo(N) (1)

The application of this procedure for all values of r, s and t will result in a set of n MOLS of order N, defined by φ_r,u,t_. The pseudo code used for the construction of MOLS is given below.

loop r = 1, n

loop s = 1, N

loop t = 1, N

u = {(t-1)*(r) + (s-1)}modulo(N) + 1

φ (r,u,t) = θ (r,s)

end loop t,s,r

This code is an implementation of Equation 1. Here the index r specifies the Latin square (or torsion angle) and runs from 1 to 2. The various steps involved in the MOLS algorithm are given in Figure S3.

1. Finney DJ (1955) Experimental design and its statistical basis*.* Cambridge University Press: London. pp. 45-67.
