# Supplementary figures and images for: An Estimate of the Numbers and Density of Low-Energy Structures (or Decoys) in the Conformational Landscape of Proteins
Source: PLoS One. 2009 Apr 9;4(4):e5148. doi: 10.1371/journal.pone.0005148 (PMC2663821; doi:10.1371/journal.pone.0005148)

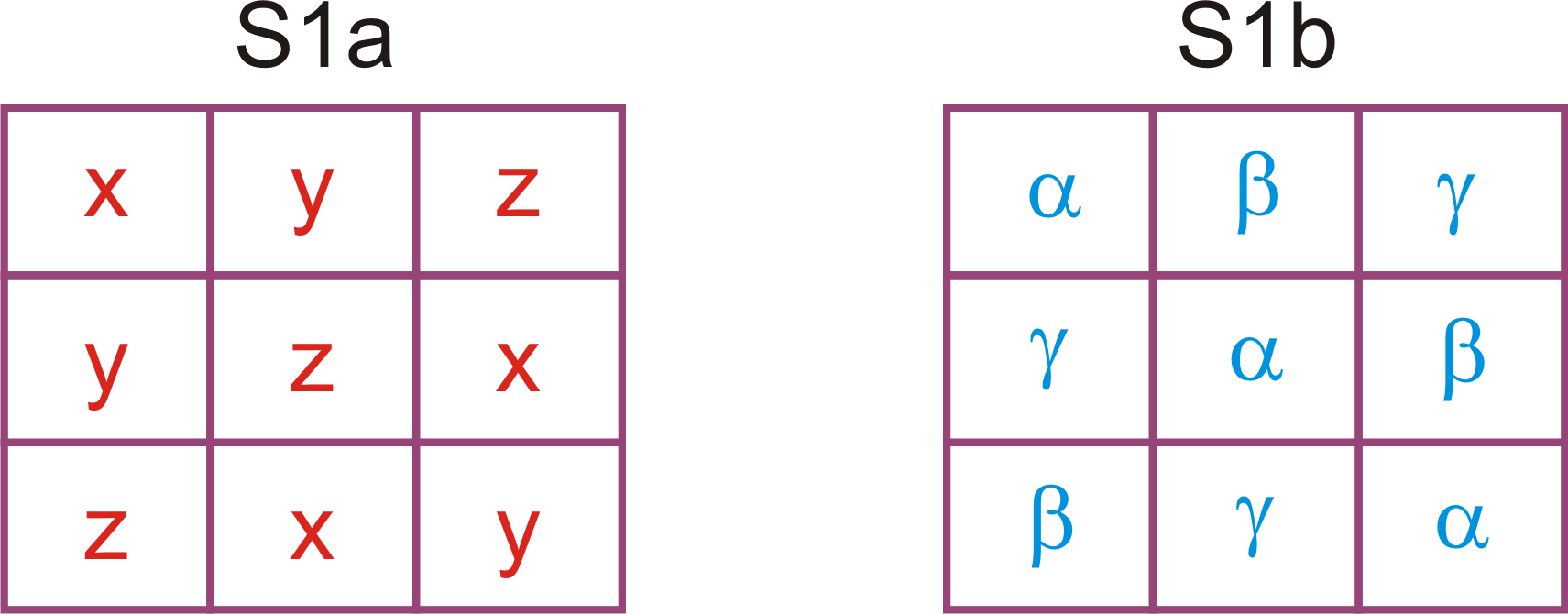

Supplement: Figure S1 — A Latin square of order 3. (a) The Latin alphabets a, b, and c and (b) the Greek alphabets α, β and γ are used as symbols for the construction of the Latin squares. (0.15 MB DOC) [file pone.0005148.s002.doc]

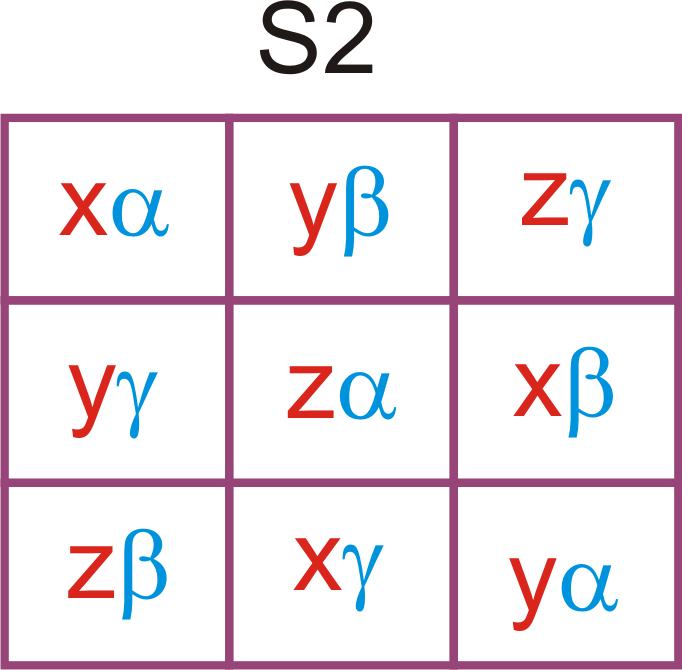

Supplement: Figure S2 — Two mutually orthogonal Latin squares (MOLS) of order 3. This is obtained by the super position of the two Latin squares given in Figures ‘S1a’ and ‘S1b’. Note that every symbol of the first square occurs once, and exactly once, with every symbol of the second square. (0.08 MB TIF) [file pone.0005148.s003.tif]

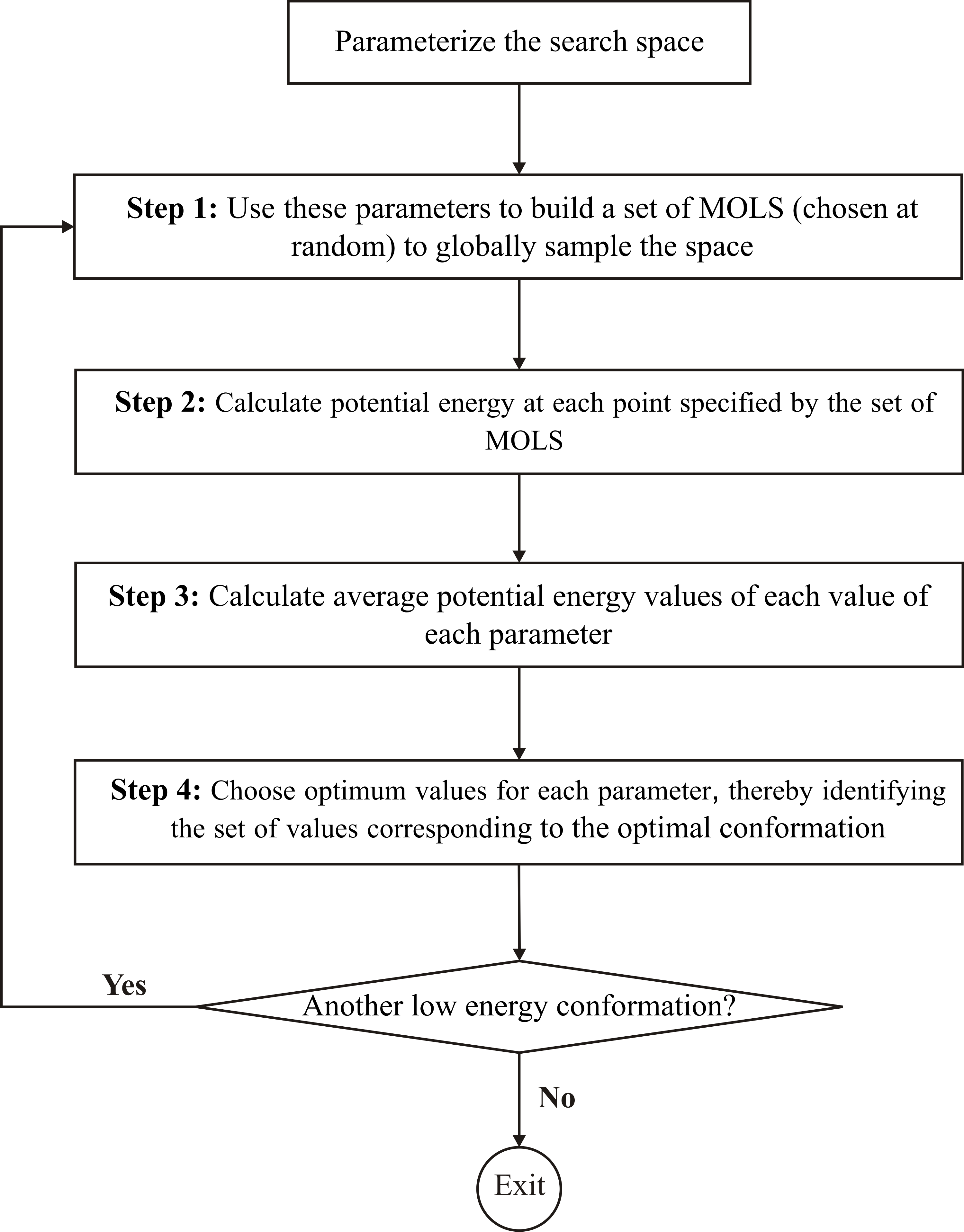

Supplement: Figure S3 — Flowchart of the MOLS algorithm. (0.95 MB TIF) [file pone.0005148.s004.tif]
